# 5-Anilino-3-benzyl­sulfanyl-6-(3-chloro­anilino)-1-phenyl-1*H*-pyrazolo­[3,4-*d*]pyrimidin-4(5*H*)-one

**DOI:** 10.1107/S1600536812037683

**Published:** 2012-09-08

**Authors:** Hong-Qing Wang, Fang-Fang He, Xiao-Feng Wang, Wei-Ping Zhou, Zhao-Jie Liu

**Affiliations:** a School of Chemistry and Chemical Engneering, University of South China, Hengyang 421001, People’s Republic of China; b College of Mathematics and Physical, University of South China, Hunan 421001, People’s Republic of China; c Institute of Organic Synthesis, Central China Normal University, Wuhan 430079, People’s Republic of China

## Abstract

In the title compound, C_30_H_23_ClN_6_OS, the benzyl, the 3-chloro­anilino, the phenyl and the anilino groups form dihedral angles of 85.95 (6), 29.63 (7), 28.55 (1) and 87.48 (6)°, respectively, with the pyrazolo­[3,4-*d*]pyrimidine unit [maximum deviation = 0.052 (2) Å]. An intra­molecular N—H⋯N hydrogen bond occurs. The crystal structure features N—H⋯O hydrogen bonds.

## Related literature

For similar compounds, see: Wang *et al.* (2004 ▶, 2008 ▶). For their applications, see: Bendich *et al.* (1954 ▶); Ballell *et al.* (2007 ▶); Holla *et al.* (2006 ▶). For standard bond lengths, see: Allen *et al.* (1987 ▶).
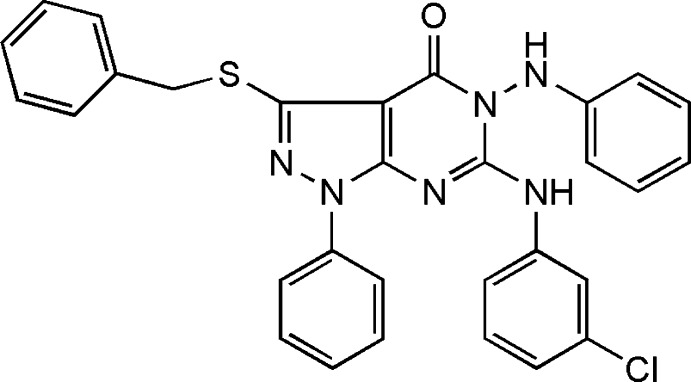


## Experimental

### 

#### Crystal data

C_30_H_23_ClN_6_OS
*M* *_r_* = 551.05
Triclinic, 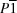

*a* = 10.881 (5) Å
*b* = 10.948 (4) Å
*c* = 12.496 (5) Å
α = 103.824 (7)°
β = 109.725 (7)°
γ = 93.377 (7)°
*V* = 1344.7 (9) Å^3^
*Z* = 2
Mo *K*α radiation
μ = 0.26 mm^−1^
*T* = 293 K
0.42 × 0.40 × 0.36 mm

#### Data collection

Bruker SMART CCD area-detector diffractometer
Absorption correction: multi-scan (*SADABS*; Bruker 2000 ▶) *T*_min_ = 0.771, *T*_max_ = 1.000
7799 measured reflections
5440 independent reflections
3418 reflections with *I* > 2σ(*I*)
*R*_int_ = 0.022

#### Refinement

*R*[*F* ^2^ > 2σ(*F* ^2^)] = 0.046
*wR*(*F* ^2^) = 0.114
*S* = 1.01
5440 reflections
360 parameters
H atoms treated by a mixture of independent and constrained refinement
Δρ_max_ = 0.40 e Å^−3^
Δρ_min_ = −0.40 e Å^−3^

### 

Data collection: *SMART* (Bruker, 2000 ▶); cell refinement: *SAINT* (Bruker, 2000 ▶); data reduction: *SAINT*; program(s) used to solve structure: *SHELXS97* (Sheldrick, 2008 ▶); program(s) used to refine structure: *SHELXL97* (Sheldrick, 2008 ▶); molecular graphics: *SHELXTL* (Sheldrick, 2008 ▶); software used to prepare material for publication: *SHELXTL* and *Mercury* (Macrae *et al.*, 2008 ▶).

## Supplementary Material

Crystal structure: contains datablock(s) I, global. DOI: 10.1107/S1600536812037683/rn2104sup1.cif

Supplementary material file. DOI: 10.1107/S1600536812037683/rn2104Isup2.mol

Supplementary material file. DOI: 10.1107/S1600536812037683/rn2104Isup3.mol

Structure factors: contains datablock(s) I. DOI: 10.1107/S1600536812037683/rn2104Isup4.hkl

Supplementary material file. DOI: 10.1107/S1600536812037683/rn2104Isup5.cml

Additional supplementary materials:  crystallographic information; 3D view; checkCIF report

## Figures and Tables

**Table 1:** Hydrogen-bond geometry (Å, °)

*D*—H⋯*A*	*D*—H	H⋯*A*	*D*⋯*A*	*D*—H⋯*A*
N5—H5⋯N6	0.79 (2)	2.19 (2)	2.605 (3)	114 (2)
N6—H6⋯O1^i^	0.81 (2)	2.12 (2)	2.904 (3)	162 (2)

Symmetry code: (i) 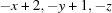.

**Fig. 1.**
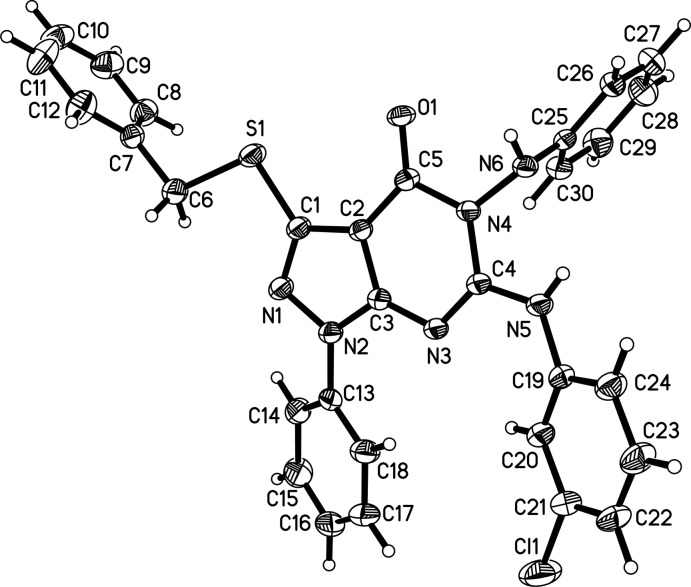
View of the title compound, with the atom-numbering scheme. Displacement ellipsoids are drawn at the 50% probability level.

**Fig. 2.**
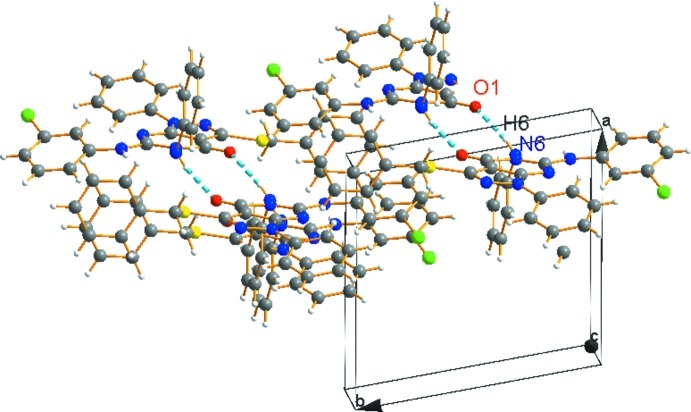
Hydrogen bonding pattern found in the title compound

## supplementary crystallographic information

### Comment

Pyrazolo[3,4-*d*]pyrimidine derivatives are important heterocycles as purine analogs(Bendich *et al.*,1954). They are usually used due to their potency as antagonists, antifungal agents (Ballell *et al.*, 2007; Holla *et al.*, 2006) and agrochemicals (Wang *et al.*, 2008). We have reported the synthesis and structure of pyrazolo[3,4-*d*]pyrimidin-4(5*H*)-one derivatives (Wang *et al.*, 2004).

The molecule and packing diagrams of the title compound were shown in Figure 1 and 2, respectively. As shown in Fig.1, the N1=C1 and N3=C4 bonds are 1.318 (3) and 1.304 (3) Å, respectively, slightly shorter than the normal C=N bond (1.329 and 1.336 Å; Allen *et al.*, 1987) but close to 1.314 (3) and 1.302 (3) Å in our previous report (Wang *et al.*, 2004), and the C2=C3 distance is 1.388 (3) Å, longer than the typical C=C(1.384 Å; Allen *et al.*, 1987). In the molecule, the N–C bonds are remarkably shorter than the normal N–C single bond(1.469 Å) and longer than the C=N double bond. The packing was stabilized by the intermolecular N–H···O hydrogen bond.

### Experimental

To a solution of iminophosphorane (2 mmol) in dry dichloromethane (20 ml) aryl isocyanate(2 mmol) was added under nitrogen atmosphere at room temperature. After the reaction mixture was stirred for 1.5 h, 0.231 g (2.0 mmol) *m*-chlorophenyl isocyanate was added, and the resulting mixture was stirred for an additional 30 min. Then the solvent was removed under reduced pressure, and 25 ml anhydrous ethanol and 1.5 ml of sodium ethoxide (3 mol/*L*) in ethanol were added to the mixture. After 3 h of stirring at room temperature, the solution was concentrated under reduced pressure and successively cooled. The crude product was collected by filtration. After recrystallization from DMF/petroleum ether or column chromatography on silica gel, white crystals were obtained.

### Refinement

H atoms were included in their idealized positions, with C—H = 0.96 Å, and refined using a riding model with *U*_iso_(H) = 1.2 *U*_eq_(C) for other H atoms.

### Crystal data

**Table:**

C_30_H_23_ClN_6_OS	*Z* = 2
*M**_r_* = 551.05	*F*(000) = 572
Triclinic, *P*1	*D*_x_ = 1.361 Mg m^−^^3^
*a* = 10.881 (5) Å	Mo *K*α radiation, λ = 0.71073 Å
*b* = 10.948 (4) Å	Cell parameters from 809 reflections
*c* = 12.496 (5) Å	θ = 2.6–25.6°
α = 103.824 (7)°	µ = 0.26 mm^−^^1^
β = 109.725 (7)°	*T* = 293 K
γ = 93.377 (7)°	Block, white
*V* = 1344.7 (9) Å^3^	0.42 × 0.40 × 0.36 mm

### Data collection

**Table:**

Bruker SMART CCD area-detector diffractometer	5440 independent reflections
Radiation source: fine-focus sealed tube	3418 reflections with *I* > 2σ(*I*)
Graphite monochromator	*R*_int_ = 0.022
phi and ω scans	θ_max_ = 26.4°, θ_min_ = 1.9°
Absorption correction: multi-scan (*SADABS*; Bruker 2000)	*h* = −13→9
*T*_min_ = 0.771, *T*_max_ = 1.000	*k* = −13→13
7799 measured reflections	*l* = −9→15

### Refinement

**Table:**

Refinement on *F*^2^	Primary atom site location: structure-invariant direct methods
Least-squares matrix: full	Secondary atom site location: difference Fourier map
*R*[*F*^2^ > 2σ(*F*^2^)] = 0.046	Hydrogen site location: inferred from neighbouring sites
*wR*(*F*^2^) = 0.114	H atoms treated by a mixture of independent and constrained refinement
*S* = 1.01	*w* = 1/[σ^2^(*F*_o_^2^) + (0.0437*P*)^2^ + 0.3561*P*] where *P* = (*F*_o_^2^ + 2*F*_c_^2^)/3
5440 reflections	(Δ/σ)_max_ = 0.001
360 parameters	Δρ_max_ = 0.40 e Å^−^^3^
0 restraints	Δρ_min_ = −0.40 e Å^−^^3^

### Special details

**Table:**

Geometry. All e.s.d.'s (except the e.s.d. in the dihedral angle between two l.s. planes) are estimated using the full covariance matrix. The cell e.s.d.'s are taken into account individually in the estimation of e.s.d.'s in distances, angles and torsion angles; correlations between e.s.d.'s in cell parameters are only used when they are defined by crystal symmetry. An approximate (isotropic) treatment of cell e.s.d.'s is used for estimating e.s.d.'s involving l.s. planes.
Refinement. Refinement of *F*^2^ against ALL reflections. The weighted *R*-factor *wR* and goodness of fit *S* are based on *F*^2^, conventional *R*-factors *R* are based on *F*, with *F* set to zero for negative *F*^2^. The threshold expression of *F*^2^ > σ(*F*^2^) is used only for calculating *R*-factors(gt) *etc*. and is not relevant to the choice of reflections for refinement. *R*-factors based on *F*^2^ are statistically about twice as large as those based on *F*, and *R*- factors based on ALL data will be even larger.

### Fractional atomic coordinates and isotropic or equivalent isotropic displacement parameters (Å^2^)

**Table:**

	*x*	*y*	*z*	*U*_iso_*/*U*_eq_	
Cl1	0.58922 (10)	−0.29645 (7)	−0.00499 (9)	0.0950 (3)	
S1	0.84719 (7)	0.64314 (5)	0.33369 (6)	0.0557 (2)	
N1	0.7368 (2)	0.41189 (16)	0.32750 (16)	0.0462 (5)	
N2	0.71298 (19)	0.28629 (16)	0.25760 (16)	0.0422 (5)	
N3	0.75420 (19)	0.16942 (15)	0.08617 (15)	0.0395 (4)	
N4	0.84282 (18)	0.30693 (15)	0.00045 (15)	0.0359 (4)	
N5	0.8107 (2)	0.08909 (17)	−0.07736 (18)	0.0448 (5)	
H5	0.844 (2)	0.108 (2)	−0.119 (2)	0.045 (7)*	
N6	0.8716 (2)	0.31275 (17)	−0.10020 (17)	0.0382 (5)	
H6	0.944 (2)	0.354 (2)	−0.0795 (18)	0.034 (6)*	
O1	0.89538 (16)	0.52346 (13)	0.07639 (13)	0.0454 (4)	
C1	0.7947 (2)	0.47893 (19)	0.27824 (19)	0.0417 (5)	
C2	0.8102 (2)	0.40058 (18)	0.17656 (18)	0.0374 (5)	
C3	0.7579 (2)	0.27820 (19)	0.16756 (18)	0.0371 (5)	
C4	0.8006 (2)	0.18745 (18)	0.00674 (18)	0.0359 (5)	
C5	0.8535 (2)	0.42237 (19)	0.08658 (18)	0.0360 (5)	
C6	0.8389 (3)	0.6721 (2)	0.4802 (2)	0.0571 (7)	
H6A	0.7510	0.6403	0.4744	0.068*	
H6B	0.9018	0.6285	0.5272	0.068*	
C7	0.8708 (2)	0.8136 (2)	0.5377 (2)	0.0438 (6)	
C8	0.7750 (3)	0.8901 (2)	0.5120 (2)	0.0568 (7)	
H8	0.6906	0.8537	0.4584	0.068*	
C9	0.8016 (3)	1.0192 (3)	0.5641 (3)	0.0661 (8)	
H9	0.7353	1.0691	0.5462	0.079*	
C10	0.9247 (3)	1.0737 (3)	0.6416 (3)	0.0691 (8)	
H10	0.9423	1.1608	0.6779	0.083*	
C11	1.0216 (3)	1.0016 (3)	0.6661 (3)	0.0770 (9)	
H11	1.1063	1.0395	0.7181	0.092*	
C12	0.9953 (3)	0.8704 (3)	0.6138 (2)	0.0655 (8)	
H12	1.0626	0.8214	0.6307	0.079*	
C13	0.6338 (2)	0.1923 (2)	0.27856 (19)	0.0421 (5)	
C14	0.5503 (3)	0.2301 (2)	0.3367 (2)	0.0567 (7)	
H14	0.5454	0.3160	0.3624	0.068*	
C15	0.4731 (3)	0.1394 (3)	0.3569 (2)	0.0700 (8)	
H15	0.4168	0.1650	0.3969	0.084*	
C16	0.4787 (3)	0.0121 (3)	0.3187 (2)	0.0657 (8)	
H16	0.4275	−0.0482	0.3335	0.079*	
C17	0.5606 (3)	−0.0250 (2)	0.2585 (2)	0.0613 (7)	
H17	0.5632	−0.1112	0.2306	0.074*	
C18	0.6397 (3)	0.0644 (2)	0.2385 (2)	0.0537 (6)	
H18	0.6962	0.0386	0.1986	0.064*	
C19	0.7924 (2)	−0.04243 (19)	−0.08586 (19)	0.0414 (5)	
C20	0.7077 (3)	−0.0968 (2)	−0.0437 (2)	0.0488 (6)	
H20	0.6589	−0.0476	−0.0069	0.059*	
C21	0.6971 (3)	−0.2271 (2)	−0.0578 (2)	0.0554 (7)	
C22	0.7651 (3)	−0.3030 (2)	−0.1140 (2)	0.0629 (8)	
H22	0.7567	−0.3900	−0.1219	0.075*	
C23	0.8462 (3)	−0.2473 (2)	−0.1585 (2)	0.0628 (7)	
H23	0.8912	−0.2978	−0.1989	0.075*	
C24	0.8620 (3)	−0.1173 (2)	−0.1443 (2)	0.0532 (6)	
H24	0.9185	−0.0804	−0.1734	0.064*	
C25	0.7690 (2)	0.34949 (17)	−0.18724 (18)	0.0367 (5)	
C26	0.7993 (3)	0.3826 (2)	−0.2774 (2)	0.0466 (6)	
H26	0.8847	0.3840	−0.2777	0.056*	
C27	0.7010 (3)	0.4132 (2)	−0.3663 (2)	0.0621 (8)	
H27	0.7206	0.4345	−0.4271	0.075*	
C28	0.5748 (3)	0.4127 (3)	−0.3663 (2)	0.0661 (8)	
H28	0.5097	0.4340	−0.4264	0.079*	
C29	0.5450 (3)	0.3804 (2)	−0.2768 (2)	0.0599 (7)	
H29	0.4597	0.3804	−0.2763	0.072*	
C30	0.6417 (2)	0.3480 (2)	−0.1879 (2)	0.0454 (6)	
H30	0.6210	0.3250	−0.1283	0.054*	

### Atomic displacement parameters (Å^2^)

**Table:**

	*U*^11^	*U*^22^	*U*^33^	*U*^12^	*U*^13^	*U*^23^
Cl1	0.1326 (8)	0.0500 (4)	0.1256 (7)	−0.0044 (4)	0.0769 (6)	0.0270 (5)
S1	0.0814 (5)	0.0344 (3)	0.0551 (4)	−0.0089 (3)	0.0419 (4)	−0.0015 (3)
N1	0.0592 (13)	0.0348 (10)	0.0453 (11)	−0.0037 (9)	0.0270 (10)	0.0030 (9)
N2	0.0562 (13)	0.0326 (10)	0.0416 (11)	−0.0012 (9)	0.0257 (10)	0.0072 (8)
N3	0.0549 (12)	0.0273 (9)	0.0405 (10)	0.0031 (8)	0.0231 (9)	0.0092 (8)
N4	0.0485 (12)	0.0268 (9)	0.0376 (10)	0.0031 (8)	0.0220 (9)	0.0095 (8)
N5	0.0684 (15)	0.0268 (9)	0.0482 (12)	0.0058 (9)	0.0333 (11)	0.0095 (9)
N6	0.0462 (13)	0.0320 (10)	0.0425 (11)	0.0015 (9)	0.0247 (10)	0.0097 (8)
O1	0.0586 (11)	0.0292 (8)	0.0553 (10)	−0.0024 (7)	0.0322 (8)	0.0093 (7)
C1	0.0506 (15)	0.0322 (11)	0.0431 (13)	−0.0009 (10)	0.0233 (11)	0.0042 (10)
C2	0.0441 (14)	0.0295 (11)	0.0390 (12)	−0.0009 (9)	0.0190 (10)	0.0060 (9)
C3	0.0446 (14)	0.0326 (11)	0.0362 (12)	0.0020 (10)	0.0175 (10)	0.0099 (9)
C4	0.0435 (14)	0.0272 (10)	0.0385 (12)	0.0043 (9)	0.0163 (10)	0.0100 (9)
C5	0.0364 (13)	0.0297 (11)	0.0419 (12)	0.0019 (9)	0.0160 (10)	0.0078 (9)
C6	0.081 (2)	0.0429 (14)	0.0478 (15)	−0.0009 (13)	0.0318 (14)	0.0046 (11)
C7	0.0543 (16)	0.0395 (12)	0.0403 (13)	0.0015 (11)	0.0266 (12)	0.0035 (10)
C8	0.0528 (17)	0.0561 (16)	0.0603 (16)	0.0026 (13)	0.0232 (13)	0.0114 (13)
C9	0.074 (2)	0.0498 (16)	0.085 (2)	0.0168 (15)	0.0418 (18)	0.0178 (15)
C10	0.087 (2)	0.0399 (14)	0.087 (2)	−0.0006 (16)	0.0523 (19)	0.0005 (14)
C11	0.061 (2)	0.0630 (19)	0.083 (2)	−0.0084 (16)	0.0222 (17)	−0.0132 (16)
C12	0.0561 (18)	0.0586 (17)	0.0715 (19)	0.0141 (14)	0.0196 (15)	0.0030 (14)
C13	0.0484 (15)	0.0421 (12)	0.0358 (12)	−0.0045 (11)	0.0161 (11)	0.0122 (10)
C14	0.0707 (19)	0.0505 (15)	0.0511 (15)	−0.0058 (13)	0.0332 (14)	0.0051 (12)
C15	0.076 (2)	0.077 (2)	0.0628 (18)	−0.0123 (16)	0.0422 (16)	0.0110 (15)
C16	0.074 (2)	0.0634 (18)	0.0602 (17)	−0.0176 (15)	0.0256 (15)	0.0226 (14)
C17	0.077 (2)	0.0452 (14)	0.0671 (18)	−0.0042 (14)	0.0309 (16)	0.0218 (13)
C18	0.0669 (18)	0.0450 (14)	0.0590 (16)	0.0046 (12)	0.0314 (14)	0.0204 (12)
C19	0.0525 (15)	0.0284 (11)	0.0404 (13)	0.0054 (10)	0.0145 (11)	0.0079 (10)
C20	0.0653 (17)	0.0320 (12)	0.0514 (14)	0.0056 (11)	0.0260 (13)	0.0089 (11)
C21	0.0706 (19)	0.0365 (13)	0.0586 (16)	−0.0001 (12)	0.0230 (14)	0.0150 (12)
C22	0.086 (2)	0.0288 (12)	0.0727 (19)	0.0135 (13)	0.0263 (17)	0.0151 (13)
C23	0.075 (2)	0.0366 (13)	0.0789 (19)	0.0184 (13)	0.0331 (16)	0.0098 (13)
C24	0.0639 (18)	0.0365 (13)	0.0624 (16)	0.0082 (12)	0.0292 (14)	0.0103 (12)
C25	0.0520 (15)	0.0214 (10)	0.0363 (12)	0.0018 (9)	0.0186 (11)	0.0046 (9)
C26	0.0625 (17)	0.0385 (12)	0.0442 (14)	0.0022 (11)	0.0292 (13)	0.0083 (11)
C27	0.088 (2)	0.0571 (16)	0.0436 (15)	0.0016 (15)	0.0250 (15)	0.0192 (13)
C28	0.075 (2)	0.0660 (18)	0.0525 (17)	0.0097 (16)	0.0115 (15)	0.0254 (14)
C29	0.0521 (17)	0.0617 (17)	0.0612 (17)	0.0069 (13)	0.0148 (14)	0.0173 (14)
C30	0.0495 (16)	0.0426 (13)	0.0473 (14)	0.0013 (11)	0.0214 (12)	0.0140 (11)

### Geometric parameters (Å, º)

**Table:**

Cl1—C21	1.746 (3)	C12—H12	0.9300
S1—C1	1.748 (2)	C13—C14	1.367 (3)
S1—C6	1.816 (2)	C13—C18	1.383 (3)
N1—C1	1.318 (3)	C14—C15	1.386 (3)
N1—N2	1.397 (2)	C14—H14	0.9300
N2—C3	1.357 (3)	C15—C16	1.373 (4)
N2—C13	1.430 (3)	C15—H15	0.9300
N3—C4	1.304 (3)	C16—C17	1.369 (4)
N3—C3	1.357 (3)	C16—H16	0.9300
N4—C4	1.389 (2)	C17—C18	1.387 (3)
N4—N6	1.409 (2)	C17—H17	0.9300
N4—C5	1.420 (3)	C18—H18	0.9300
N5—C4	1.351 (3)	C19—C20	1.376 (3)
N5—C19	1.415 (3)	C19—C24	1.392 (3)
N5—H5	0.79 (2)	C20—C21	1.388 (3)
N6—C25	1.426 (3)	C20—H20	0.9300
N6—H6	0.81 (2)	C21—C22	1.369 (4)
O1—C5	1.223 (2)	C22—C23	1.375 (4)
C1—C2	1.421 (3)	C22—H22	0.9300
C2—C3	1.388 (3)	C23—C24	1.384 (3)
C2—C5	1.420 (3)	C23—H23	0.9300
C6—C7	1.508 (3)	C24—H24	0.9300
C6—H6A	0.9700	C25—C30	1.381 (3)
C6—H6B	0.9700	C25—C26	1.393 (3)
C7—C12	1.369 (4)	C26—C27	1.382 (4)
C7—C8	1.377 (3)	C26—H26	0.9300
C8—C9	1.375 (4)	C27—C28	1.372 (4)
C8—H8	0.9300	C27—H27	0.9300
C9—C10	1.357 (4)	C28—C29	1.379 (4)
C9—H9	0.9300	C28—H28	0.9300
C10—C11	1.351 (4)	C29—C30	1.382 (3)
C10—H10	0.9300	C29—H29	0.9300
C11—C12	1.396 (4)	C30—H30	0.9300
C11—H11	0.9300		
			
C1—S1—C6	101.17 (11)	C14—C13—C18	120.3 (2)
C1—N1—N2	105.43 (17)	C14—C13—N2	119.3 (2)
C3—N2—N1	110.80 (16)	C18—C13—N2	120.4 (2)
C3—N2—C13	129.75 (18)	C13—C14—C15	119.5 (2)
N1—N2—C13	118.85 (17)	C13—C14—H14	120.2
C4—N3—C3	113.84 (17)	C15—C14—H14	120.2
C4—N4—N6	117.28 (16)	C16—C15—C14	120.9 (3)
C4—N4—C5	123.97 (17)	C16—C15—H15	119.6
N6—N4—C5	118.71 (15)	C14—C15—H15	119.6
C4—N5—C19	128.2 (2)	C17—C16—C15	119.1 (2)
C4—N5—H5	115.2 (17)	C17—C16—H16	120.4
C19—N5—H5	115.4 (17)	C15—C16—H16	120.4
N4—N6—C25	114.04 (18)	C16—C17—C18	120.8 (3)
N4—N6—H6	109.4 (15)	C16—C17—H17	119.6
C25—N6—H6	114.9 (15)	C18—C17—H17	119.6
N1—C1—C2	111.45 (18)	C13—C18—C17	119.3 (2)
N1—C1—S1	123.62 (16)	C13—C18—H18	120.4
C2—C1—S1	124.93 (16)	C17—C18—H18	120.4
C3—C2—C5	119.40 (19)	C20—C19—C24	120.6 (2)
C3—C2—C1	105.10 (18)	C20—C19—N5	123.2 (2)
C5—C2—C1	135.25 (19)	C24—C19—N5	116.2 (2)
N3—C3—N2	125.70 (18)	C19—C20—C21	118.1 (2)
N3—C3—C2	127.08 (19)	C19—C20—H20	120.9
N2—C3—C2	107.21 (18)	C21—C20—H20	120.9
N3—C4—N5	121.72 (19)	C22—C21—C20	122.6 (2)
N3—C4—N4	123.69 (19)	C22—C21—Cl1	119.16 (19)
N5—C4—N4	114.59 (19)	C20—C21—Cl1	118.2 (2)
O1—C5—C2	128.6 (2)	C21—C22—C23	118.3 (2)
O1—C5—N4	119.93 (19)	C21—C22—H22	120.9
C2—C5—N4	111.51 (17)	C23—C22—H22	120.9
C7—C6—S1	108.09 (16)	C22—C23—C24	121.1 (2)
C7—C6—H6A	110.1	C22—C23—H23	119.4
S1—C6—H6A	110.1	C24—C23—H23	119.4
C7—C6—H6B	110.1	C23—C24—C19	119.2 (2)
S1—C6—H6B	110.1	C23—C24—H24	120.4
H6A—C6—H6B	108.4	C19—C24—H24	120.4
C12—C7—C8	118.1 (2)	C30—C25—C26	119.7 (2)
C12—C7—C6	121.6 (2)	C30—C25—N6	122.8 (2)
C8—C7—C6	120.3 (2)	C26—C25—N6	117.5 (2)
C9—C8—C7	121.3 (3)	C27—C26—C25	119.2 (2)
C9—C8—H8	119.3	C27—C26—H26	120.4
C7—C8—H8	119.3	C25—C26—H26	120.4
C10—C9—C8	119.9 (3)	C28—C27—C26	121.0 (2)
C10—C9—H9	120.1	C28—C27—H27	119.5
C8—C9—H9	120.1	C26—C27—H27	119.5
C11—C10—C9	120.1 (3)	C27—C28—C29	119.8 (3)
C11—C10—H10	120.0	C27—C28—H28	120.1
C9—C10—H10	120.0	C29—C28—H28	120.1
C10—C11—C12	120.3 (3)	C28—C29—C30	120.0 (3)
C10—C11—H11	119.9	C28—C29—H29	120.0
C12—C11—H11	119.9	C30—C29—H29	120.0
C7—C12—C11	120.3 (3)	C25—C30—C29	120.3 (2)
C7—C12—H12	119.9	C25—C30—H30	119.8
C11—C12—H12	119.9	C29—C30—H30	119.8
			
C1—N1—N2—C3	0.7 (3)	C6—C7—C8—C9	179.4 (2)
C1—N1—N2—C13	−171.3 (2)	C7—C8—C9—C10	0.7 (4)
C4—N4—N6—C25	100.4 (2)	C8—C9—C10—C11	1.1 (4)
C5—N4—N6—C25	−77.3 (2)	C9—C10—C11—C12	−1.3 (5)
N2—N1—C1—C2	0.0 (3)	C8—C7—C12—C11	2.0 (4)
N2—N1—C1—S1	−179.17 (17)	C6—C7—C12—C11	−179.6 (2)
C6—S1—C1—N1	13.9 (2)	C10—C11—C12—C7	−0.3 (5)
C6—S1—C1—C2	−165.1 (2)	C3—N2—C13—C14	−149.5 (2)
N1—C1—C2—C3	−0.6 (3)	N1—N2—C13—C14	20.7 (3)
S1—C1—C2—C3	178.52 (17)	C3—N2—C13—C18	29.3 (4)
N1—C1—C2—C5	173.3 (2)	N1—N2—C13—C18	−160.5 (2)
S1—C1—C2—C5	−7.5 (4)	C18—C13—C14—C15	1.0 (4)
C4—N3—C3—N2	178.3 (2)	N2—C13—C14—C15	179.8 (2)
C4—N3—C3—C2	−3.2 (3)	C13—C14—C15—C16	−0.4 (4)
N1—N2—C3—N3	177.6 (2)	C14—C15—C16—C17	−0.9 (4)
C13—N2—C3—N3	−11.5 (4)	C15—C16—C17—C18	1.6 (4)
N1—N2—C3—C2	−1.1 (3)	C14—C13—C18—C17	−0.3 (4)
C13—N2—C3—C2	169.7 (2)	N2—C13—C18—C17	−179.1 (2)
C5—C2—C3—N3	7.2 (4)	C16—C17—C18—C13	−1.0 (4)
C1—C2—C3—N3	−177.7 (2)	C4—N5—C19—C20	30.2 (4)
C5—C2—C3—N2	−174.1 (2)	C4—N5—C19—C24	−152.2 (2)
C1—C2—C3—N2	1.0 (3)	C24—C19—C20—C21	2.0 (4)
C3—N3—C4—N5	176.6 (2)	N5—C19—C20—C21	179.5 (2)
C3—N3—C4—N4	−3.8 (3)	C19—C20—C21—C22	−1.5 (4)
C19—N5—C4—N3	−9.2 (4)	C19—C20—C21—Cl1	−179.75 (19)
C19—N5—C4—N4	171.1 (2)	C20—C21—C22—C23	−0.4 (4)
N6—N4—C4—N3	−170.6 (2)	Cl1—C21—C22—C23	177.8 (2)
C5—N4—C4—N3	6.9 (3)	C21—C22—C23—C24	1.8 (4)
N6—N4—C4—N5	9.1 (3)	C22—C23—C24—C19	−1.3 (4)
C5—N4—C4—N5	−173.4 (2)	C20—C19—C24—C23	−0.7 (4)
C3—C2—C5—O1	175.9 (2)	N5—C19—C24—C23	−178.3 (2)
C1—C2—C5—O1	2.6 (5)	N4—N6—C25—C30	−15.6 (3)
C3—C2—C5—N4	−3.7 (3)	N4—N6—C25—C26	167.26 (17)
C1—C2—C5—N4	−177.0 (2)	C30—C25—C26—C27	−0.1 (3)
C4—N4—C5—O1	177.8 (2)	N6—C25—C26—C27	177.1 (2)
N6—N4—C5—O1	−4.7 (3)	C25—C26—C27—C28	0.6 (4)
C4—N4—C5—C2	−2.6 (3)	C26—C27—C28—C29	−0.4 (4)
N6—N4—C5—C2	174.89 (19)	C27—C28—C29—C30	−0.4 (4)
C1—S1—C6—C7	−175.34 (19)	C26—C25—C30—C29	−0.7 (3)
S1—C6—C7—C12	−96.8 (3)	N6—C25—C30—C29	−177.8 (2)
S1—C6—C7—C8	81.5 (3)	C28—C29—C30—C25	0.9 (4)
C12—C7—C8—C9	−2.2 (4)		

### Hydrogen-bond geometry (Å, º)

**Table:**

*D*—H···*A*	*D*—H	H···*A*	*D*···*A*	*D*—H···*A*
N5—H5···N6	0.79 (2)	2.19 (2)	2.605 (3)	114 (2)
N6—H6···O1^i^	0.81 (2)	2.12 (2)	2.904 (3)	162 (2)

Symmetry code: (i) −*x*+2, −*y*+1, −*z*.
